# Instrumental Variable Estimation of the Causal Effect of Plasma 25-Hydroxy-Vitamin D on Colorectal Cancer Risk: A Mendelian Randomization Analysis

**DOI:** 10.1371/journal.pone.0037662

**Published:** 2012-06-06

**Authors:** Evropi Theodoratou, Tom Palmer, Lina Zgaga, Susan M. Farrington, Paul McKeigue, Farhat V. N. Din, Albert Tenesa, George Davey-Smith, Malcolm G. Dunlop, Harry Campbell

**Affiliations:** 1 Centre for Population Health Sciences, University of Edinburgh, Edinburgh, United Kingdom; 2 MRC Human Genetics Unit, Colon Cancer Genetics Group and Academic Coloproctology, Institute of Genetics and Molecular Medicine, University of Edinburgh, Western General Hospital Edinburgh, United Kingdom; 3 MRC Centre for Causal Analyses in Translational Epidemiology, School of Social and Community Medicine, University of Bristol, Bristol, United Kingdom; 4 Andrija Stampar School of Public Health, Medical School, University of Zagreb, Zagreb, Croatia; 5 The Roslin Institute, The University of Edinburgh, Easter Bush, Roslin, Scotland; University of Hong Kong, Hong Kong

## Abstract

Vitamin D deficiency has been associated with several common diseases, including cancer and is being investigated as a possible risk factor for these conditions. We reported the striking prevalence of vitamin D deficiency in Scotland. Previous epidemiological studies have reported an association between low dietary vitamin D and colorectal cancer (CRC). Using a case-control study design, we tested the association between plasma 25-hydroxy-vitamin D (25-OHD) and CRC (2,001 cases, 2,237 controls). To determine whether plasma 25-OHD levels are causally linked to CRC risk, we applied the control function instrumental variable (IV) method of the Mendelian randomization (MR) approach using four single nucleotide polymorphisms (rs2282679, rs12785878, rs10741657, rs6013897) previously shown to be associated with plasma 25-OHD. Low plasma 25-OHD levels were associated with CRC risk in the crude model (odds ratio (OR): 0.76, 95% Confidence Interval (CI): 0.71, 0.81, p: 1.4×10^−14^) and after adjusting for age, sex and other confounding factors. Using an allele score that combined all four SNPs as the IV, the estimated causal effect was OR 1.16 (95% CI 0.60, 2.23), whilst it was 0.94 (95% CI 0.46, 1.91) and 0.93 (0.53, 1.63) when using an upstream (rs12785878, rs10741657) and a downstream allele score (rs2282679, rs6013897), respectively. 25-OHD levels were inversely associated with CRC risk, in agreement with recent meta-analyses. The fact that this finding was not replicated when the MR approach was employed might be due to weak instruments, giving low power to demonstrate an effect (<0.35). The prevalence and degree of vitamin D deficiency amongst individuals living in northerly latitudes is of considerable importance because of its relationship to disease. To elucidate the effect of vitamin D on CRC cancer risk, additional large studies of vitamin D and CRC risk are required and/or the application of alternative methods that are less sensitive to weak instrument restrictions.

## Introduction

**Figure 1:**
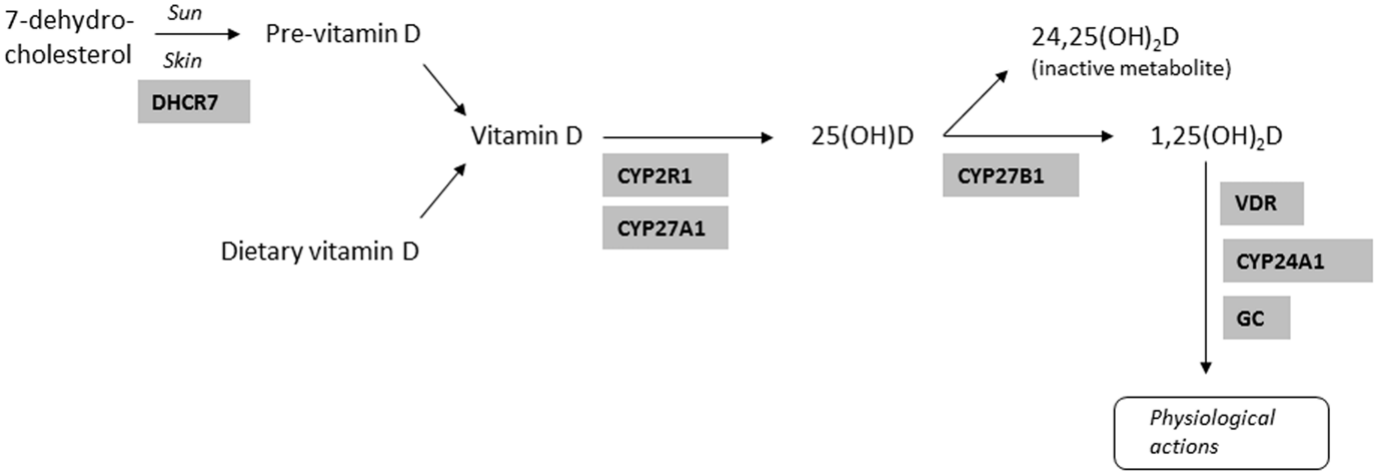
Vitamin D metabolic pathway.

Vitamin D can be ingested or synthesized in the skin from inactive precursors through the action of UV sunlight. Its active form, 1,25(OH)_2_D (1,25(OH)_2_D_2_ and/or 1,25(OH)_2_D_3_) is produced after two hydroxylation steps in the liver and kidneys (**Figure 1**) [1]. The prevalence of vitamin D deficiency in Scotland is high due to high northern latitude, often cloudy weather (lack of sunlight impairs vitamin D synthesis during winter months), indoors oriented lifestyle and poor diet, and so routine vitamin D and calcium supplementation for the housebound (>65 years old) is recommended [2]. In a recent study of over 2000 healthy individuals living in Scotland, we found that 77.5% of the individuals were vitamin D deficient [3]. Although the Reference Nutrient Intake (RNI) of vitamin D by the Scientific Advisory Committee on Nutrition in Scotland for people over 65 years old is 10 ug per day [4], there is a great variation of the recommended daily allowances (RDA) by different research groups and institutions [5]–[8].

Vitamin D has been considered relevant to skeletal disease and calcium metabolism, but there is growing evidence that vitamin D deficiency might be a risk factor for cancer, cardiovascular, metabolic, infectious and autoimmune diseases [3]. In particular, vitamin D may affect colorectal cancer (CRC) risk via its binding to the vitamin D receptor (VDR) [9] influencing cell proliferation, differentiation, apoptosis and angiogenesis [10], [11] or affecting insulin resistance [12]. Results from case-control and cohort studies are inconclusive, but results from cohort studies measuring 25-hydroxy-vitamin D (25-OHD) in the blood or the serum are more consistent indicating an inverse association with CRC [13]–[15].

**Figure 2:**
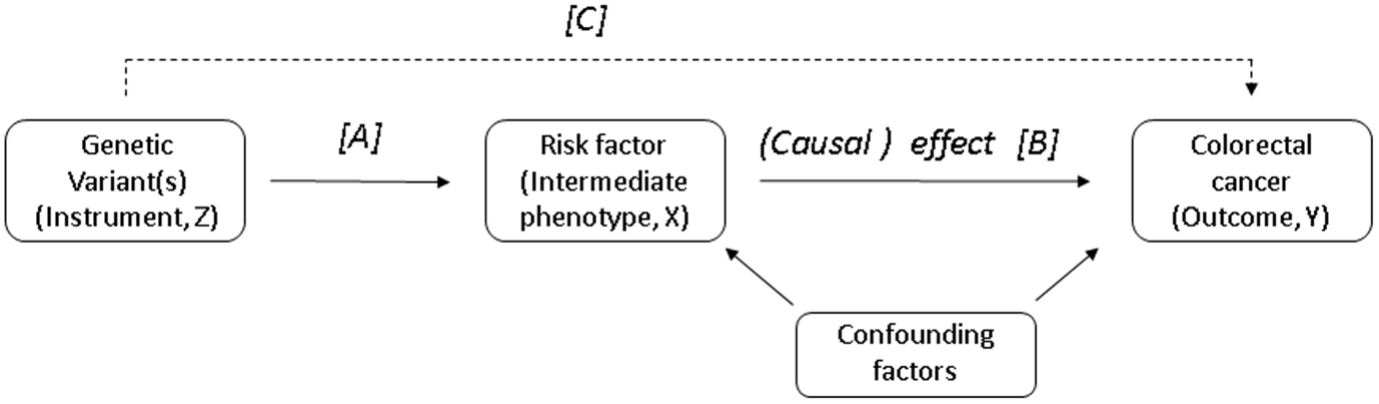
Directed acyclic graph (DAG) showing the instrumental variable assumptions underpinning our Mendelian randomisation study (note the instrument is not allowed to have a direct effect on the outcome, hence this line is dashed). Instrumental variable models use associations C and A to estimate the causal effect of a risk factor on an outcome (B).

Establishing causal relationships between environmental exposures and common diseases using conventional methods of observational studies is problematic due to unresolved confounding, reverse causation and selection bias [16]. The theory underpinning the Mendelian randomization (MR) approach is based on the random assortment of alleles at the time of gamete formation, which is equivalent to a randomized controlled trial in which people are randomly allocated to therapeutic interventions. The main concept of a MR study is based on three relationships: genotype–intermediate phenotype; intermediate phenotype–disease; genotype–disease [17], [18] and it can be used to identify causal environmental risk factors without the several potential problems of observational epidemiology [19]. The MR approach can also strengthen causal conclusions by limiting reverse causation problems (biological, through exposure assignment, due to reporting bias), selection bias and regression dilution bias [19]. **Figure 2** illustrates how this concept is applied to inform causal inference.

The analytic approach employed here for MR is the instrumental variable (IV) model, in which the genetic variant is treated as an instrument which is assumed to be associated with the disease only through its association with the intermediate phenotype [18]. This requires firstly the identification of one or more genetic variants (typically a single nucleotide polymorphism or SNP) as the IV that is known from published data to be associated with the phenotype [18]. The three key assumptions underlying the MR approach are: a) the genotype is associated with the phenotype; b) the genotype is independent of measured and unmeasured confounders; and c) that the effect of genotype on outcome is mediated only through the intermediate phenotype (no pleiotropy) [17], [18].

In this study, we set out to evaluate the relationship between CRC, plasma 25-OHD levels and genotype at 4 genetic loci tagging genes involved in vitamin D metabolism (**Table S1**) and which have previously been shown to be associated with plasma vitamin D levels in a pooled meta-analysis of Genome Wide Association Studies [20]. In order to estimate whether there is a causal relationship between plasma 25-OHD and CRC risk we applied the control function IV estimator.

## Methods

### Ethics Statement

Ethical approval for the SOCCS study was obtained from the MultiCentre Research Ethics committee for Scotland (reference number 01/0/05) and from the Research and Development Office of NHS Lothian (reference number 2003/W/GEN/05).

### Study Population

We studied a subset of 2,001 cases and 2,237 controls from a case-control study of CRC (Study Of Colorectal Cancer in Scotland, SOCCS). We aimed to recruit all incident cases (1999–2006) of adenocarcinoma of colorectum presenting to surgical units in Scotland (18–79 years old). Exclusions were patient death before ascertainment, patient too ill to participate, recurrent cases, or patient unable to give informed consent due to learning difficulties or other medical conditions. We recruited about 40% of all incident cases in Scotland over the study period. During the same period controls were drawn randomly from a population-based register (community health index) and invited to participate. Participation rates among those approached were approximately 58% for cases and an estimated 57% for controls. More than 99% of the study participants were white Caucasian (see [21] for further recruitment details).

The subjects completed one questionnaire with lifestyle and cancer information and they were asked to report their status one year prior diagnosis or recruitment, including information about their general medical history, physical activity, smoking status, intake of any regular intake of aspirin and NSAIDs, height, weight and waist circumference were recorded. Additionally a semi-quantitative food frequency questionnaire (Scottish Collaborative Group FFQ, Version 6.41) was completed by participants (http://www.foodfrequency.org), which consisted of 150 foods and the individuals were asked to describe the amount and frequency of each food on the list they have eaten a year prior to diagnosis or recruitment. Further information about the questionnaires were presented in detail previously [21]. In addition, each recruited cancer subject was assigned an American Joint Committee on Cancer (AJCC) stage derived from a synthesis of clinical, pathological and imaging information [22]. Finally, for a subset of the cancer cases (1,423, of those 1,376 with 25-OHD measured) there was information about the symptoms they had developed before recruitment. We grouped the cases into four categories: (1) no symptoms (190 cases), (2) mild symptoms (290; including: change in bowel habit, constipation, intermittent diarrhoea and constipation, more frequent stools, diarrhoea, loose stools, excess wind, mucus in stool and abdominal discomfort) and (3) severe symptoms (220; including rectal bleeding, vomiting, weight loss, loss of energy, loss of appetite and nausea) and (4) both, mild and severe symptoms (676).

Family history risk was determined according to the Scottish Executive cancer guidelines (http://www.sehd.scot.nhs.uk/), The criteria for high family history risk of colorectal cancer are: 1) at least three family members affected by colorectal cancer or at least two with colorectal cancer and one with endometrial cancer in at least two generations; one affected relative must be <50 years old at diagnosis and one of the relatives must be a first degree relative of the other two; or 2) presence of the HNPCC syndrome; or 3) untested first degree relatives of known gene carriers. The criteria for moderate risk are: 1) one first degree relative affected by colorectal cancer when aged <45 years old; or 2) two affected first degree relatives with one aged <55 years old; or 3) three affected relatives with colorectal or endometrial cancer, who are first degree relatives of each other and one a first degree relative of the consultant. Individuals that do not fulfil all the above criteria are classified as low family history risk (Scottish Executive cancer guidelines). For this analysis family history was coded as low vs. medium/high family history of CRC.

### Measurement of Plasma 25-OHD

The liquid chromatography-tandem mass spectrometry (LC-MS/MS) method was used to measure 25-hydroxyvitamin D3 and D2. This paper presents the 25-OHD (the total of 25-OHD2 and 25-OHD3); however, most of our samples contained no D2 (<3 ng/ml). The lower limit of detection with the LC-MS/MS method was 4 ng/ml for D3 [23]. The LC MS/MS method was performed following standard protocols and appropriate quality control procedures (including multiple measurements of the same sample from our cohort and standardization against standard reference material, SRM 972) and it has been rated as the preferred 25-OHD measurement method for population studies by an international panel of experts [23]. More details about this method can be found elsewhere [23], [24]. For the analysis 25-OHD measurements were standardized to remove the prominent effect of the month when blood was taken on the 25-OHD concentration, as described in detail in Zgaga et al [3].

### Genotyping Data

DNA samples were accurately quantified by Pico-GreenTM and quality controlled prior to dispatch. Genotyping was undertaken using TaqMan in the Wellcome Trust Clinical Research Facility (WTCRF) in Edinburgh. 2,000 subjects for the rs2282679 were genotyped as part of an array-based candidate gene approach, using the Illumina Infinium I Custom array platform and performed by Illumina (San Diego). Case and control DNA samples were stored, genotyped and analysed in the same way. In addition to avoid potential systematic batch-to-batch variation or bias, samples were anonymised as to affection status and were randomly distributed within plates. Data were subject to Illumina or WTCRF quality control procedures. Assumptions of Hardy-Weinberg Equilibrium (HWE) were tested using a chi-squared test.

### Statistical Analysis

The statistical package used was Stata version 11.0 (Stata Corp, College Station, Texas). Participants were divided into quintiles based on the combined distributions of cases and controls. Logistic regression models were used to estimate the strength of association between CRC risk and vitamin D plasma levels. The associations were tested in three logistic regression models (crude model, model I and model II). Model I was corrected for age and sex and Model II was corrected for age, sex, Carstairs Deprivation Index, energy (MJoules/day), smoking (non-smoker, former smoker and current smoker), body mass index (BMI, kg/m^2^, continuous), regular NSAID intake (yes vs. no), family history (low vs. medium/high) of cancer and physical activity (hours of cycling and other sports activities, 4 groups). We also tested the association after sex, stage of cancer at diagnosis (AJCC), presence of symptoms and time between diagnosis and recruitment stratification. In addition the association between CRC and rs2282679, rs12785878, rs10741657 and rs6013897 was tested. Dataset for this analysis was larger, and it comprised all SOCCS study participants for whom genotyping of selected SNPs was successful (up to 5,449). We also tested the interaction between genotype and vitamin D plasma levels on CRC by comparing a model with and without an interaction term between the two variables, using a likelihood ratio test. Assumptions of Hardy-Weinberg Equilibrium were tested using a chi-squared test.

To estimate the causal odds ratio we applied the control function IV estimator for a 3-level categorical instrument Z coded 0, 1, 2 (SNP) a continuous intermediate phenotype X (plasma 25-OHD_3_) and a binary outcome Y (CRC). The first stage of the control function is a linear regression of the intermediate phenotype (X) on the instrument(s) (Z), which generates predicted values for the intermediate phenotype. The second stage is a logistic regression of the outcome (Y) on the predicted values of the intermediate phenotype including the estimated residuals from the first-stage linear regression in the second-stage logistic regression [25]. The rationale is that the first-stage residuals may be correlated with unmeasured confounding factors. In addition to a crude model, we also adjusted for age and sex. The strength of the applied instruments were evaluated using the F statistics from the first stage linear regression, with values lower than 10 taken as evidence of a weak instrument [26]. Finally, we applied four additional IV estimators which are presented and described in the supplementary material (**Methods S1**).

## Results

### Levels of Plasma 25-OHD and CRC

Colorectal cancer risk was associated with lower levels of plasma 25-OHD in the crude model (Odds ratio (OR): 0.76, 95% Confidence Interval (CI): 0.71, 0.81, p: 1.4×10^−14^), after adjusting for age and sex (OR: 0.75, 95% CI: 0.70, 0.81, p: 9.1×10^−15^) and after adjusting for age, sex, Carstairs Deprivation Index, energy, smoking, BMI, regular NSAID intake, family history of cancer and physical activity (OR: 0.75, 95% CI: 0.69, 0.81, p: 4.6×10^−12^) (**Table 1**). The 25-OHD CRC association was stronger for men (crude model: OR: 0.68, 95% CI: 0.62, 0.75, p: 1.1×10^−13^) than women (crude model: OR: 0.84, 95% CI: 0.76, 0.93, p: 0.0009) (**Table S2**). The 25-OHD CRC association was similar for early (crude model: OR: 0.79, 95% CI: 0.72, 0.86, p: 2.0×10^−8^) versus late AJCC stage (crude model: OR: 0.74, 95% CI: 0.68, 0.80, p: 5.8×10^−12^; **Table S3**). Furthermore, the 25-OHD CRC association was weaker for those CRC patients that had no symptoms at the time of the diagnosis (crude model: OR: 0.87, 95% CI: 0.73, 1.02, p: 0.09), when compared to those with mild (crude model: OR: 0.80, 95% CI: 0.70, 0.91, p: 0.001) or severe symptoms (crude model: OR: 0.78, 95% CI: 0.67, 0.90, p: 0.001; **Table S4**). Finally, the 25-OHD CRC association was similar for those CRC patients that were recruited soon after diagnosis (crude model: OR: 0.77, 95% CI: 0.71, 0.83, p: 7.8×10^−5^), when compared to those who were recruited later (crude model: OR: 0.76, 95% CI: 0.70, 0.83, p: 1.3×10^−10^; **Table S5**).

**Table 1:** Logistic regression analysis for the association between plasma 25-0HD on colorectal cancer risk.

	N		Crude model			Model I*			Model II†		
Standard logistic regression analysis	*Cases*	*Controls*	*OR*	*95% CI*	*p-value*	*OR*	*95% CI*	*p-value*	*OR*	*95% CI*	*p-value*
25-OHD (continuous; ng/ml)	2001	2237	0.76	0.71, 0.81	1.4×10^−14^	0.75	0.70, 0.81	9.13×10^−15^	0.75	0.69, 0.81	4.6×10^−12^
25-0HD (binary)											
<10 ng/ml	973	826	1.00			1.00			1.00		
≥10 ng/ml	1028	1411	0.62	0.55, 0.70	1.2×10^−14^	0.61	0.54, 0.69	7.1×10^−15^	0.62	0.54, 0.72	6.1×10^−11^
25-0HD (quintiles)											
<5.31	486	366	1.00			1.00			1.00		
5.31–9.39	461	425	0.82	0.68, 0.99	0.04	0.82	0.68, 0.99	0.04	0.75	0.61, 0.94	0.01
9.39–13.20	376	430	0.66	0.54, 0.80	<0.0005	0.66	0.54, 0.80	<0.0005	0.67	0.53, 0.83	<0.0005
13.20–18.36	358	505	0.53	0.44, 0.65	<0.0005	0.53	0.44, 0.64	<0.0005	0.49	0.39, 0.61	<0.0005
≥18.36	320	511	0.47	0.39, 0.57	<0.0005	0.46	0.38, 0.56	<0.0005	0.45	0.35, 0.56	<0.0005
* p-value trend*					2.2×10^−18^			7.8×10^−19^			3.6×10^−15^

* Adjusted for age and sex.

† Adjusted for age, sex, Carstairs Deprivation Index, energy (MJoules/ day), smoking (non-smoker, former smoker and current smoker), body mass index (BMI, kg/m^2^, continuous), regular Non Steroidal Anti-Inflammatory Drug (NSAID) intake (yes vs. no), family history of cancer and physical activity (hours of cycling and other sports activities, 4 groups).

### Genotype and Plasma 25-OHD Levels

There was no evidence for departure from HWE for all four SNPs: rs2282679 p-value = 0.25, rs12785878 p-value = 0.78, rs10741657 p-value = 0.07 and rs6013897 p-value = 0.52. The A allele of rs2282679 and the T allele of rs12785878 were associated with higher levels of plasma 25-OHD (**Table 2**). In particular, we found that rs2282679 and rs12785878 genotypes were associated with a decreased risk of 25-OHD deficiency defined as <10 ng/ml (rs2282679: for each A allele OR = 0.88, 95% CI 0.80, 0.98, p = 0.02; rs12785878: for each T allele OR = 0.89, 95% CI 0.79, 1.00, p = 0.05; rs10741657; **Table 2**). These associations were not different when we restricted the analysis only in the controls (data not shown).

**Table 2:** Association between plasma 25-0HD levels and rs2282679, rs12785878, rs10741657 and rs6013897.

	25-0HD levels		Crude model			Model I*		
Standard logistic regression analysis	*<10 ng/ml*	*≥10 ng/ml*	*OR*	*95% CI*	*p-value*	*OR*	*95% CI*	*p-value*
*rs2282679*								
CC	133	165	1.00			1.00		
AC	698	905	0.96	0.75, 1.23	0.73	0.95	0.74, 1.22	0.69
AA	767	1163	0.82	0.64, 1.05	0.11	0.80	0.63, 1.03	0.09
per A allele			0.88	0.80, 0.98	0.02	0.87	0.79, 0.97	0.01
*rs12785878*								
GG	72	66	1.00			1.00		
TG	470	620	0.69	0.49, 0.99	0.04	0.69	0.48, 0.99	0.04
TT	980	1366	0.65	0.47, 0.93	0.02	0.65	0.46, 0.92	0.02
per T allele			0.89	0.79, 1.00	0.05	0.89	0.79, 1.00	0.05
*rs10741657*								
GG	512	672	1.00			1.00		
GA	385	925	0.97	0.84, 1.13	0.71	0.97	0.83, 1.13	0.71
AA	211	318	0.87	0.71, 1.07	0.19	0.88	0.71, 1.08	0.22
per A allele			0.94	0.84, 1.04	0.23	0.94	0.85, 1.04	0.25
*rs6013897*								
AA	47	72	1.00			1.00		
TA	470	599	1.20	0.82, 1.77	0.35	1.19	0.82, 1.75	0.39
TT	968	1319	1.12	0.77, 1.64	0.54	1.11	0.76, 1.62	0.60
per T allele			0.98	0.87, 1.11	0.73	0.98	0.86, 1.10	0.70

* Adjusted for age and sex.

### Genotype and CRC

Overall there was no evidence of an association between any of the four SNPs and CRC risk (**Table 3**). When we stratified according to plasma 25-OHD levels, the rs10741657 SNP was associated with a decreased CRC risk for those of low plasma 25-OHD levels (per A allele OR = 0.88, 95% CI 0.75, 1.02, p = 0.09) and with an increased CRC risk for those of high plasma 25-OHD levels (OR = 1.12, 95% CI 0.98, 1.27, p = 0.09), with a p-value of interaction (p = 0.05).

**Table 3:** Association between colorectal cancer risk and rs2282679, rs12785878, rs10741657 and rs6013897.

Standard logistic regression analysis*	Case/control status		Mean (SD) of25-0HD (ng/ml)		Crude model			Model I^†^		
*All-samples*	*Cases*	*Controls*	*Cases*	*Controls*	*OR*	*95% CI*	*p-value*	*OR*	*95% CI*	*p-value*
*rs2282679*										
CC	216	218	11.63 (7.45)	12.59 (8.45)	1.00			1.00		
AC	1130	1160	11.01 (7.82)	13.29 (8.24)	0.98	0.80, 1.21	0.87	0.99	0.81, 1.21	0.92
AA	1325	1400	12.00 (7.86)	14.01 (8.65)	0.96	0.78, 1.17	0.66	0.96	0.78, 1.17	0.68
per A allele					0.97	0.90, 1.06	0.55	0.97	0.90, 1.06	0.55
*rs12785878*										
GG	81	112	9.16 (7.29)	10.67 (7.11)	1.00			1.00		
TG	749	798	10.89 (7.50)	13.50 (8.20)	1.30	0.96, 1.76	0.09	1.30	0.96, 1.76	0.09
TT	1667	1713	11.69 (7.72)	13.59 (8.59)	1.35	1.00, 1.80	0.05	1.35	1.00, 1.81	0.05
per T allele					1.08	0.98, 1.20	0.11	1.08	0.98, 1.20	0.11
*rs10741657*										
GG	851	855	11.15 (7.58)	13.82 (8.88)	1.00			1.00		
GA	1082	1201	11.54 (7.88)	13.19 (8.25)	0.91	0.80, 1.03	0.12	0.90	0.79, 1.02	0.11
AA	402	371	11.82 (7.62)	13.78 (8.51)	1.09	0.92, 1.29	0.33	1.08	0.91, 1.29	0.35
per A allele					1.01	0.94, 1.10	0.72	1.01	0.93, 1.10	0.77
*rs6013897*										
AA	92	83	12.44 (8.83)	13.89 (11.98)	1.00			1.00		
TA	739	806	11.45 (7.29)	12.84 (8.44)	0.83	0.60, 1.13	0.24	0.84	0.61, 1.15	0.27
TT	1615	1648	11.32 (7.83)	13.72 (8.34)	0.88	0.65, 1.20	0.43	0.90	0.66, 1.21	0.48
per T allele					1.02	0.92, 1.13	0.71	1.02	0.92, 1.13	0.68
*Allele score*	2815	2924			1.00	0.97, 1.04	0.87	1.01	0.97, 1.04	0.69
*“Synthesis” allele score*	2630	2761			1.04	0.98, 1.10	0.17	1.04	0.99, 1.10	0.14
*“Metabolism” allele score*	2779	2884			0.99	0.94, 1.05	0.79	0.99	0.94, 1.05	0.82
***<10ng/ml 25-0HD levels***	***Cases***	***Controls***			***OR***	***95% CI***	***p-value***	***OR***	***95% CI***	***p-value***
*rs2282679*										
CC	66	67	6.01 (2.84)	5.71 (3.05)	1.00			1.00		
AC	392	306	5.21 (2.90)	5.54 (2.97)	1.30	0.90, 1.89	0.17	1.29	0.89, 1.88	0.17
AA	399	368	5.47 (2.78)	5.81 (2.79)	1.10	0.76, 1.59	0.61	1.10	0.76, 1.59	0.61
per A allele					0.96	0.82, 1.12	0.58	0.96	0.82, 1.12	0.60
*rs12785878*										
GG	32	40	3.98 (2.79)	5.18 (3.12)	1.00			1.00		
TG	249	221	5.37 (2.87)	5.88 (2.81)	1.41	0.86, 2.32	0.18	1.41	0.86, 2.32	1.18
TT	526	454	5.52 (2.83)	5.66 (2.90)	1.45	0.89, 2.34	0.13	1.46	0.90, 2.36	0.13
per T allele					1.10	0.93, 1.31	0.27	1.11	0.93, 1.32	0.25
*rs10741657*										
GG	296	216	5.55 (2.84)	5.60 (2.99)	1.00			1.00		
GA	346	339	5.45 (2.81)	5.74 (2.85)	0.74	0.59, 0.94	0.01	0.74	0.59, 0.94	0.01
AA	113	98	5.11 (2.73)	5.61 (2.79)	0.84	0.61, 1.16	0.29	0.84	0.61, 1.17	0.31
per A allele					0.88	0.75, 1.02	0.09	0.88	0.75, 1.02	0.10
*rs6013897*										
AA	23	24	5.00 (2.62)	4.55 (2.71)	1.00			1.00		
TA	237	233	5.66 (2.74)	5.36 (3.06)	1.06	0.58, 1.93	0.85	1.07	0.59, 1.96	0.82
TT	531	437	5.35 (2.84)	5.88 (2.78)	1.27	0.71, 2.28	0.43	1.29	0.72, 2.31	0.40
per T allele					1.17	0.97, 1.41	0.10	1.18	0.98, 1.42	0.09
***≥10 ng/ml 25-0HD levels***	***Cases***	***Controls***			***OR***	***95% CI***	***p-value***	***OR***	***95% CI***	***p-value***
*rs2282679*										
CC	69	96	17.01 (6.46)	17.34 (7.68)	1.00			1.00		
AC	380	525	17.00 (6.71)	17.80 (6.86)	1.01	0.72, 1.41	0.97	1.01	0.72, 1.42	0.94
AA	487	676	17.35 (6.53)	18.48 (7.40)	1.00	0.72, 1.39	0.99	1.00	0.72, 1.39	0.99
per A allele					1.00	0.87, 1.14	0.98	0.99	0.87, 1.14	0.93
*rs12785878*										
GG	23	43	16.36 (5.11)	15.78 (5.86)	1.00			1.00		
TG	246	374	16.47 (6.55)	18.00 (6.91)	1.23	0.72, 2.09	0.45	1.24	0.73, 2.11	0.43
TT	584	782	17.24 (6.41)	18.21 (7.34)	1.40	0.83, 2.34	0.21	1.40	0.83, 2.35	0.20
per T allele					1.15	0.98, 1.35	0.09	1.15	0.98, 1.35	0.09
*rs10741657*										
GG	271	401	17.27 (6.30)	18.26 (7.78)	1.00			1.00		
GA	383	542	17.04 (6.87)	17.85 (7.00)	1.05	0.85, 1.28	0.67	1.04	0.85, 1.27	0.70
AA	148	170	16.94 (5.99)	18.50 (6.98)	1.29	0.98, 1.69	0.07	1.28	0.98, 1.68	0.07
per A allele					1.12	0.98, 1.27	0.09	1.11	0.98, 1.27	0.10
*rs6013897*										
AA	35	37	17.33 (8.01)	19.95 (11.77)	1.00			1.00		
TA	249	350	16.96 (5.86)	17.82 (7.09)	0.75	0.46, 1.23	0.25	0.76	0.47, 1.24	0.28
TT	545	774	17.13 (6.69)	18.15 (7.07)	0.74	0.46, 1.20	0.22	0.75	0.47, 1.21	0.24
per T allele					0.94	0.80, 1.10	0.44	0.94	0.80, 1.10	0.44

Before applying the MR approach we assessed the IV assumptions. The first (that genotype is associated with the phenotype) was fulfilled since we selected four SNPs that were found to be linked to plasma 25-OHD levels in a pooled meta-analysis of Genome Wide Association Studies [20]. The second (genotype is independent of measured and unmeasured confounders) was tested by investigating whether the instruments were associated with any of the measured confounding factors that might influence the relationship between plasma 25-OHD levels and CRC (**Table S6**) and, as expected [27], there was no evidence for an association between these confounding factors and the genotypes. Finally, the third assumption (effect of genotype on outcome is mediated only through the intermediate phenotype) was tested by interrogating of pleiotropic links of genes and SNPs that we recently created [28]. For all SNPs there was no evidence of pleiotropy and they were only found to be linked to plasma vitamin D levels.

Using the rs2282679 as the IV, the estimated causal effect of plasma 25-0HD on CRC risk was 0.94 (95% CI 0.49, 1.83), and the F-statistic for the rs2282679 from the first stage of the IV analysis was 15.80 in the age and sex adjusted analysis (**Table 4**). Using the rs12785878 as the IV the causal effect was 1.23 (95% CI 0.60, 2.53), and the F-statistic for the rs12785878 from the first stage of the IV analysis was 13.50 (**Table 4**). Using the rs10741657 as the IV the causal effect was 0.89 (95% CI 0.40, 1.98), and the F-statistic for the rs10741657 from the first stage of the IV analysis was 10.89 (**Table 4**). Finally, using the rs6013897 as the IV the causal effect was 0.99 (95% CI 0.40, 2.45), and the F-statistic for the rs6013897 from the first stage of the IV analysis was 0.98 (**Table 4**). The results of the other IV estimators are presented in **Tables S7, S8, S9, and S10**.

**Table 4:** Control function instrumental variable estimator of the causal odds ratio for the effect of plasma 25(0H)D on colorectal cancer risk.

Model	plasma 25-0HD (continuous, ng/ml)		
*rs2282679*	*OR*	*95% CI*	F statistic
Unadjusted	0.58	0.11, 3.10	7.29
Adjusted for age and sex	0.94	0.49, 1.83	15.80
*rs12785878*			
Unadjusted	4.03	0.93, 17.41	9.70
Adjusted for age and sex	1.23	0.60, 2.53	13.50
*rs10741657*			
Unadjusted	1.15	0.00, 762.13	0.49
Adjusted for age and sex	0.89	0.40, 1.98	10.89
*rs6013897*			
Unadjusted	2.26	0.06, 86.31	1.58
Adjusted for age and sex	0.99	0.40, 2.45	0.98
*Allele score* *			
Unadjusted	1.50	0.42, 5.35	12.95
Adjusted for age and sex	1.16	0.60, 2.23	16.52
*Allele score (upstream)* †			
Unadjusted	1.83	0.22, 15.19	4.63
Adjusted for age and sex	0.94	0.46, 1.91	7.87
*Allele score (downstream)* ‡			
Unadjusted	0.83	0.34, 2.05	14.89
Adjusted for age and sex	0.93	0.53, 1.63	12.67

* Allele score that combines all 4 SNPs: rs12785878, rs10741657, rs2282679 and rs6013897.

† Allele score that combines 2 upstream (synthesis) SNPs: rs12785878, rs10741657.

‡ Allele score that combines 2 “metabolism” SNPs: rs2282679 and rs6013897.

Furthermore, we combined these four SNPs to form three allele scores: 1) one allele score that combined all four SNPs, 2) an upstream allele score that combined the SNPs rs12785878 and rs10741657 and 3) a downstream allele score that combined the SNPs rs2282679 and rs6013897. We then used this allele scores as the IV. The causal effect for the overall allele score was 1.16 (95% CI 0.60, 2.23; F-statistic 16.52), for the upstream allele score was 0.94 (95% CI 0.46, 1.91; F-statistic 7.87) and for the downstream allele score was 0.93 (95% CI 0.53, 1.63; F-statistic 12.67) (**Table 4**).

## Discussion

### Levels of Plasma 25-OHD and CRC

In this study, low levels of plasma 25-OHD were associated with a higher risk of CRC in the whole sample and after stratification for sex, tumour stage and severity of symptoms at presentation. These results are in accordance with two recent meta-analyses of serum or plasma prospective studies [14], [29]. In addition a systematic review and meta-analysis on colorectal adenoma (CRA) showed a decreased risk with both incidence and recurrence of CRA for an increase of 25-OHD by 20 ng/ml [30]. However, a randomised clinical trial (RCT) (2686 average risk subjects) found no effect of vitamin D supplementation and incidence of CRC [31]. Similarly, a second RCT (Women’s Health Initiative –WHI) investigating the effects of daily calcium and vitamin D supplementation for seven years showed no effect on CRC incidence among postmenopausal women [32]. However, it should be noted that neither of these RCTs were designed and powered for cancer as the primary outcome. In addition, re-analysis of the WHI RCT found that concurrent oestrogen therapy was an effect modifier of calcium and vitamin D supplementation and for women that were not assigned to oestrogen therapy calcium and vitamin D supplementation decreased CRC risk [33]. Data from case-control and cohort studies examining the associations between dietary vitamin D intake and CRC are inconclusive [13].

### Genotype, Plasma 25-OHD Levels and CRC

The A allele of rs2282679 and the T allele of rs12785878 were associated with higher levels of plasma 25-OHD. These results are in accordance with a GWAS investigating genetic determinants of vitamin D deficiency [20]. The rs2282679 SNP is located in the *GC* gene, which encodes a vitamin D binding protein that binds and transports vitamin D (**Figure 1**) [20]. The rs12785878 SNP is located in the *DHCR7* gene that encodes the enzyme 7-dehydrocholesterol (7-DHC) reductase, which converts 7-DHC to cholesterol. 7-DHC is a precursor of vitamin D_3_. Mutations in the *DHCR7* may lead to a decreased activity of the 7-DHC reductase and therefore to high levels of 7-DHC and vitamin D_3_ (**Figure 1**) [20]. However the other two SNPs (rs10741657, rs6013897) that were also found to be strongly associated with vitamin D levels in the vitamin D genome wide association study were not associated with vitamin D status in our cohort. rs10741657 is located in the *CYP2R1* gene, which encodes an enzyme thought to be involved in the 25-hydroxylation of vitamin D_3_ to 25(OHD) [20]. rs6013897 is located in the *CYP24A1* gene, which encodes an enzyme that initiates the degradation of 1,25(OH)_2_D [20]. The evidence for the role of the enzymes coded by *CYP2R1*and *CYP24A1* is limited and not replicated in other candidate studies [20].

None of the four SNPs were associated with CRC risk, although we found an interaction between 25-OHD levels and rs10741657. The results of the MR analysis did not support a causal relationship between plasma 25-OHD and CRC risk. Although not significant, the inverse relationship was noted when rs2282679 or rs10741657 were used as instrument, but not when rs12785878 or rs6013897. The results remained inconsistent when the three allele scores of the four SNPs was used. The fact that the inverse association that was observed when we applied the conventional epidemiological methods was not replicated when the MR approach was used might be due to several reasons. It is possible that unmeasured or latent variables confounded the associations and there is no true effect of vitamin D on CRC. An alternative explanation is that there might be reverse causality between vitamin D and CRC, given that the plasma of the cases was collected after diagnosis: it cannot be excluded that low plasma 25-OHD is the consequence of disease or a result of patients being bedbound and lacking the exposure to sun. However, when we looked at cases with very mild or no symptoms or cases at the very early stages of the disease we still observed an inverse association between 25-OHD levels and CRC. In addition, given the biological potential of vitamin D having a causal link with cancer, factors that affect the performance of the IV estimators might also explain these findings.

Major limitations of conventional instrumental variable approaches result from the strict assumptions that need to be satisfied for method to be reliable. It is true that genotype is associated with phenotype, however, SNPs that have been used as instruments are only weakly associated to phenotype and explain only a small portion of trait variance. While we tested for common confounders, it is possible that hidden confounding from unmeasured variables affects the analysis. While we see no association between genotype and the outcome, weaker pleiotropic links cannot be excluded with certainty.

We are currently working on new instrumental variable methods for assessing causality between 25-OHD concentrations and CRC, based on the platform for the Bayesian analysis of complex statistical models using Markov chain Monte Carlo methods. This method improves on the classical Mendelian Randomisation approach as it allows for pleiotropic links between components of the model, enables easy inclusion of other covariates and does not depend as much on the strength of the instruments.

The wide application of the genome wide association studies (GWAS), has allowed for the MR studies to become more feasible now, as common SNPs linked to various intermediate phenotypes have been identified [34]. To date in 2011, several applied MR studies were published investigating causal relationships in a wide range of diseases including cancer [35], coronary heart disease [36], [37], diabetes [38], [39], mental disorders[40]–[42], lung function [43] and other diseases [44], [45]. Some of these studies replicated the results of the conventional epidemiological methods and confirmed causality (or no causality) of the intermediate phenotype on the outcome [40], [44], [45]. One of the main reasons behind this lack of replication is the fact that the genetic instruments that are employed are generally weak and therefore the power of the MR studies will be inadequate [34].

According to a recent study on the power and sample size requirements of MR studies based on the strength of the instruments, found that MR studies will require large (n>1000) and often very large (n>10000) sample sizes to draw causal conclusions that are statistically significant [34]. Based on their findings from simulated analyses most of the published MR studies are under-powered (see **Table S11** for a review of the 11 MR studies published in 2011). Using simulations presented in the paper of Pierce et al [34] and accounting for sample size, strength of the employed instruments and observed effect size, we estimate that the sample size in this study gave power of ∼0.35.

### Conclusion

This study shows that higher plasma 25-OHD levels are associated with a lower CRC risk, a finding that is consistent with recent meta-analyses of prospective studies. However, this finding was not replicated when the MR approach was employed. This finding might be due to a lack of a true effect of vitamin D on CRC or due to reverse causation. It may also be due to weak instruments and limited statistical power. The lack of power is a common characteristic of many MR studies and therefore a careful selection of instruments plus an adequate sample size are deemed necessary for this method to be able to make causal conclusions. Given the extent of vitamin D deficiency among individuals living in high latitudes, a large consortium of similar vitamin D and CRC studies and/or the application of alternative methods that are less sensitive to weak instrument bias [46] are necessary to refine the effect of vitamin D on CRC cancer and other chronic diseases.

## Supporting Information

Table S1 **Information about the SNPs that were used as Instrumental variables in the MR analysis.**
(DOC)

Table S2 **Logistic regression analysis for the association between plasma 25-0HD on colorectal cancer risk after sex stratification.**
(DOC)

Table S3 **Logistic regression analysis for the association between plasma 25-0HD on colorectal cancer risk after stage stratification.**
(DOC)

Table S4 **Logistic regression analysis for the association between plasma 25-0HD on colorectal cancer risk after stratification for presence of symptoms.**
(DOC)

Table S5 **Logistic regression analysis for the association between plasma 25-0HD on colorectal cancer risk after stratification based on the time between diagnosis and recruittment (TDR).**
(DOC)

Table S6 **Distribution of possible confounding factors and the instruments.**
(DOC)

Table S7 **Wald/ratio instrumental variable estimator of the causal odds ratio for the effect of plasma 25(0H)D on colorectal cancer risk.**
(DOC)

Table S8 **Two stage instrumental variable estimator of the causal odds ratio for the effect of plasma 25(0H)D on colorectal cancer risk.**
(DOC)

Table S9 **Multiplicative structural mean models instrumental variable estimator of the causal odds ratio for the effect of plasma 25(0H)D on colorectal cancer risk.**
(DOC)

Table S10 **Logistic structural mean models instrumental variable estimator of the causal odds ratio for the effect of plasma 25(0H)D on colorectal cancer risk.**
(DOC)

Table S11 **Review of the characteristics of the MR studies published in 2011.**
(DOC)

Methods S1 **Information about the following Instrumental Variable estimators: Wald (ratio) estimator, Two stage least squares estimator, Multiplicative structural mean models, Logistic structural mean models.**
(DOC)
