# Supplementary material for: Instrumental Variable Estimation of the Causal Effect of Plasma 25-Hydroxy-Vitamin D on Colorectal Cancer Risk: A Mendelian Randomization Analysis
Source: PLoS One. 2012 Jun 6;7(6):e37662. doi: 10.1371/journal.pone.0037662 (PMC3368918; doi:10.1371/journal.pone.0037662)
Supplement: Table S1 — Information about the SNPs that were used as Instrumental variables in the MR analysis. (DOC) [file pone.0037662.s001.doc]

Supplementary Table S1: Information about the SNPs that were used as Instrumental variables in the MR analysis

| SNP | Gene | Function |
| --- | --- | --- |
| rs2282679 | *GC* | Encodes a vitamin D binding protein that binds and transports vitamin D |
| rs12785878 | *DHCR7* | Encodes the enzyme 7-dehydrocholesterol (7-DHC) reductase, which converts 7-DHC to cholesterol |
| rs10741657 | *CYP2R1* | Encodes an enzyme thought to be involved in the 25-hydroxylation of vitamin D3 to 25(OHD) |
| rs6013897 | *CYP24A1* | Encodes an enzyme that initiates the degradation of 1,25(OH)2D |
