# Supplementary material for: Instrumental Variable Estimation of the Causal Effect of Plasma 25-Hydroxy-Vitamin D on Colorectal Cancer Risk: A Mendelian Randomization Analysis
Source: PLoS One. 2012 Jun 6;7(6):e37662. doi: 10.1371/journal.pone.0037662 (PMC3368918; doi:10.1371/journal.pone.0037662)
Supplement: Table S2 — Logistic regression analysis for the association between plasma 25-0HD on colorectal cancer risk after sex stratification. (DOC) [file pone.0037662.s002.doc]

Supplementary Table S2: Logistic regression analysis for the association between plasma 25-0HD on colorectal cancer risk after sex stratification

| **Standard logistic**  **regression analysis** | **N** | | **Crude model** | | | **Model I** | | | **Model II** | | |
| --- | --- | --- | --- | --- | --- | --- | --- | --- | --- | --- | --- |
| ***Males*** | *Cases* | *Controls* | *OR* | *95% CI* | *p-value* | *OR* | *95% CI* | *p-value* | *OR* | *95% CI* | *p-value* |
| 25-OHD  (continuous; ng/ml) | 1130 | 1256 | 0.68 | 0.62, 0.75 | 1.1x10^-13^ | 0.68 | 0.62, 0.75 | 1.0x10^-13^ | 0.69 | 0.61, 0.77 | 6.9x10^-11^ |
| 25-0HD (binary) |  |  |  |  |  |  |  |  |  |  |  |
| <10ng/ml | 562 | 433 | 1.00 |  |  | 1.00 |  |  | 1.00 |  |  |
| ≥10ng/ml | 568 | 823 | 0.53 | 0.45, 0.63 | 7.9x10^-14^ | 0.53 | 0.45, 0.63 | 6.0x10^-14^ | 0.53 | 0.44, 0.64 | 5.5x10^-11^ |
| 25-0HD (quintiles) |  |  |  |  |  |  |  |  |  |  |  |
| <1.67 | 271 | 181 | 1.00 |  |  | 1.00 |  |  | 1.00 |  |  |
| 1.67-2.24 | 274 | 232 | 0.79 | 0.61, 1.02 | 0.07 | 0.80 | 0.62, 1.03 | 0.08 | 0.77 | 0.58, 1.03 | 0.07 |
| 2.24-2.58 | 217 | 259 | 0.56 | 0.43, 0.73 | <0.0005 | 0.57 | 0.44, 0.73 | <0.0005 | 0.55 | 0.41, 0.74 | <0.0005 |
| 2.58-2.91 | 198 | 305 | 0.43 | 0.33, 0.56 | <0.0005 | 0.43 | 0.33, 0.56 | <0.0005 | 0.41 | 0.30, 0.55 | <0.0005 |
| ≥2.91 | 170 | 279 | 0.41 | 0.31, 0.53 | <0.0005 | 0.40 | 0.31, 0.53 | <0.0005 | 0.41 | 0.30, 0.55 | <0.0005 |
| *p-value trend* |  |  |  |  | 1.2x10^-15^ |  |  | 6.4x10^-16^ |  |  | 1.3x10^-12^ |
| ***Females*** | *Cases* | *Controls* | *OR* | *95% CI* | *p-value* | *OR* | *95% CI* | *p-value* | *OR* | *95% CI* | *p-value* |
| 25-OHD  (continuous; ng/ml) | 871 | 980 | 0.84 | 0.76, 0.93 | 0.0009 | 0.84 | 0.76, 0.93 | 0.001 | 0.83 | 0.73, 0.93 | 0.002 |
| 25-0HD (binary) |  |  |  |  |  |  |  |  |  |  |  |
| <10ng/ml | 413 | 394 | 1.00 |  |  | 1.00 |  |  | 1.00 |  |  |
| ≥10ng/ml | 458 | 588 | 0.74 | 0.62, 0.89 | 0.001 | 0.74 | 0.61, 0.89 | 0.002 | 0.76 | 0.61, 0.95 | 0.02 |
| 25-0HD (quintiles) |  |  |  |  |  |  |  |  |  |  |  |
| <1.67 | 215 | 185 | 1.00 |  |  | 1.00 |  |  | 1.00 |  |  |
| 1.67-2.24 | 187 | 193 | 0.83 | 0.63, 1.10 | 0.19 | 0.83 | 0.63, 1.10 | 0.20 | 0.73 | 0.53, 1.02 | 0.07 |
| 2.24-2.58 | 159 | 171 | 0.80 | 0.60, 1.07 | 0.13 | 0.80 | 0.59, 1.07 | 0.13 | 0.86 | 0.61, 1.20 | 0.37 |
| 2.58-2.91 | 160 | 200 | 0.69 | 0.52, 0.92 | 0.01 | 0.68 | 0.51, 0.91 | 0.01 | 0.62 | 0.44, 0.87 | 0.006 |
| ≥2.91 | 150 | 232 | 0.56 | 0.42, 0.74 | <0.0005 | 0.55 | 0.41, 0.74 | <0.0005 | 0.50 | 0.35, 0.72 | <0.0005 |
| *p-value trend* |  |  |  |  | 2.9x10^-5^ |  |  | 2.8x10^-5^ |  |  | 0.0001 |
