# Supplementary material for: Instrumental Variable Estimation of the Causal Effect of Plasma 25-Hydroxy-Vitamin D on Colorectal Cancer Risk: A Mendelian Randomization Analysis
Source: PLoS One. 2012 Jun 6;7(6):e37662. doi: 10.1371/journal.pone.0037662 (PMC3368918; doi:10.1371/journal.pone.0037662)
Supplement: Table S3 — Logistic regression analysis for the association between plasma 25-0HD on colorectal cancer risk after stage stratification. (DOC) [file pone.0037662.s003.doc]

Supplementary Table S3: Logistic regression analysis for the association between plasma 25-0HD on colorectal cancer risk after stage stratification

| **Standard logistic**  **regression analysis** | **N** | | **Crude model** | | | **Model I** | | | **Model II** | | |
| --- | --- | --- | --- | --- | --- | --- | --- | --- | --- | --- | --- |
| ***AJCC stage 1, 2*** | *Cases* | *Controls* | *OR* | *95% CI* | *p-value* | *OR* | *95% CI* | *p-value* | *OR* | *95% CI* | *p-value* |
| 25-OHD  (continuous; ng/ml) | 1049 | 2237 | 0.79 | 0.72, 0.86 | 2.0x10^-8^ | 0.79 | 0.73, 0.86 | 3.4x10^-8^ | 0.79 | 0.72, 0.87 | 3.1x10^-6^ |
| 25-0HD (binary) |  |  |  |  |  |  |  |  |  |  |  |
| <10ng/ml | 503 | 829 | 1.00 |  |  | 1.00 |  |  | 1.00 |  |  |
| ≥10ng/ml | 546 | 1412 | 0.64 | 0.55, 0.74 | 2.7x10^-9^ | 0.64 | 0.55, 0.74 | 5.0x10^-9^ | 0.64 | 0.53, 0.76 | 4.1x10^-7^ |
| 25-0HD (quintiles) |  |  |  |  |  |  |  |  |  |  |  |
| <1.67 | 246 | 366 | 1.00 |  |  | 1.00 |  |  | 1.00 |  |  |
| 1.67-2.24 | 240 | 425 | 0.84 | 0.67, 1.05 | 0.13 | 0.84 | 0.67, 1.05 | 0.13 | 0.82 | 0.63, 1.07 | 0.14 |
| 2.24-2.58 | 204 | 430 | 0.71 | 0.56, 0.89 | 0.003 | 0.70 | 0.56, 0.89 | 0.003 | 0.70 | 0.53, 0.91 | 0.009 |
| 2.58-2.91 | 180 | 505 | 0.53 | 0.42, 0.67 | <0.0005 | 0.53 | 0.42, 0.67 | <0.0005 | 0.54 | 0.41, 0.70 | <0.0005 |
| ≥2.91 | 179 | 511 | 0.52 | 0.41, 0.66 | <0.0005 | 0.53 | 0.42, 0.67 | <0.0005 | 0.51 | 0.38, 0.67 | <0.0005 |
| *p-value trend* |  |  |  |  | 4.1x10^-11^ |  |  | 1.0x10^-10^ |  |  | 2.4x10^-8^ |
| ***AJCC stage 3,4*** | *Cases* | *Controls* | *OR* | *95% CI* | *p-value* | *OR* | *95% CI* | *p-value* | *OR* | *95% CI* | *p-value* |
| 25-OHD  (continuous; ng/ml) | 893 | 2237 | 0.74 | 0.68, 0.80 | 5.8x10^-12^ | 0.73 | 0.67, 0.79 | 1.0x10^-12^ | 0.72 | 0.65, 0.79 | 1.0x10^-10^ |
| 25-0HD (binary) |  |  |  |  |  |  |  |  |  |  |  |
| <10ng/ml | 440 | 829 | 1.00 |  |  | 1.00 |  |  | 1.00 |  |  |
| ≥10ng/ml | 458 | 1412 | 0.61 | 0.52, 0.71 | 6.8x10^-10^ | 0.59 | 0.51, 0.70 | 9.8x10^-11^ | 0.62 | 0.51, 0.75 | 5.0x10^-7^ |
| 25-0HD (quintiles) |  |  |  |  |  |  |  |  |  |  |  |
| <1.67 | 225 | 366 | 1.00 |  |  | 1.00 |  |  | 1.00 |  |  |
| 1.67-2.24 | 202 | 425 | 0.77 | 0.61, 0.98 | 0.03 | 0.78 | 0.61, 0.98 | 0.04 | 0.66 | 0.50, 0.88 | 0.004 |
| 2.24-2.58 | 164 | 430 | 0.62 | 0.49, 0.79 | <0.0005 | 0.61 | 0.48, 0.78 | <0.0005 | 0.63 | 0.48, 0.83 | 0.001 |
| 2.58-2.91 | 169 | 505 | 0.54 | 0.43, 0.69 | <0.0005 | 0.53 | 0.42, 0.68 | <0.0005 | 0.46 | 0.34, 0.61 | <0.0005 |
| ≥2.91 | 133 | 511 | 0.42 | 0.33, 0.54 | <0.0005 | 0.40 | 0.31, 0.52 | <0.0005 | 0.38 | 0.28, 0.52 | <0.0005 |
| *p-value trend* |  |  |  |  | 3.8x10^-13^ |  |  | 2.3x10^-14^ |  |  | 2.2x10^-11^ |
