# Supplementary material for: Instrumental Variable Estimation of the Causal Effect of Plasma 25-Hydroxy-Vitamin D on Colorectal Cancer Risk: A Mendelian Randomization Analysis
Source: PLoS One. 2012 Jun 6;7(6):e37662. doi: 10.1371/journal.pone.0037662 (PMC3368918; doi:10.1371/journal.pone.0037662)
Supplement: Table S4 — Logistic regression analysis for the association between plasma 25-0HD on colorectal cancer risk after stratification for presence of symptoms. (DOC) [file pone.0037662.s004.doc]

Supplementary Table S4: Logistic regression analysis for the association between plasma 25-0HD on colorectal cancer risk after stratification for presence of symptoms

| **Standard logistic**  **regression analysis** | **N** | | **Crude model** | | | **Model I** | | | **Model II** | | |
| --- | --- | --- | --- | --- | --- | --- | --- | --- | --- | --- | --- |
| ***No symptoms*** | *Cases* | *Controls* | *OR* | *95% CI* | *p-value* | *OR* | *95% CI* | *p-value* | *OR* | *95% CI* | *p-value* |
| 25-OHD  (continuous; ng/ml) | 190 | 2237 | 0.87 | 0.73, 1.02 | 0.09 | 0.87 | 0.73, 1.02 | 0.09 | 0.88 | 0.72, 1.09 | 0.24 |
| 25-0HD (binary) |  |  |  |  |  |  |  |  |  |  |  |
| <10ng/ml | 88 | 829 | 1.00 |  |  | 1.00 |  |  | 1.00 |  |  |
| ≥10ng/ml | 102 | 1412 | 0.68 | 0.51, 0.92 | 0.01 | 0.68 | 0.51, 0.92 | 0.01 | 0.74 | 0.52, 1.06 | 0.10 |
| 25-0HD (quintiles) |  |  |  |  |  |  |  |  |  |  |  |
| <1.67 | 36 | 366 | 1.00 |  |  | 1.00 |  |  | 1.00 |  |  |
| 1.67-2.24 | 49 | 425 | 1.17 | 0.75, 1.84 | 0.49 | 1.16 | 0.74, 1.83 | 0.52 | 1.00 | 0.59, 1.72 | 0.99 |
| 2.24-2.58 | 44 | 430 | 1.04 | 0.66, 1.65 | 0.88 | 1.02 | 0.64, 1.63 | 0.92 | 0.94 | 0.54, 1.61 | 0.81 |
| 2.58-2.91 | 30 | 505 | 0.60 | 0.37, 1.00 | 0.05 | 0.59 | 0.36, 0.98 | 0.04 | 0.72 | 0.41, 1.26 | 0.25 |
| ≥2.91 | 31 | 511 | 0.62 | 0.37, 1.02 | 0.06 | 0.63 | 0.38, 1.03 | 0.07 | 0.58 | 0.32, 1.04 | 0.07 |
| *p-value trend* |  |  |  |  | 0.003 |  |  | 0.003 |  |  | 0.03 |
| ***Only mild symptoms*** | *Cases* | *Controls* | *OR* | *95% CI* | *p-value* | *OR* | *95% CI* | *p-value* | *OR* | *95% CI* | *p-value* |
| 25-OHD  (continuous; ng/ml) | 290 | 2237 | 0.80 | 0.70, 0.91 | 0.001 | 0.81 | 0.71, 0.94 | 0.004 | 0.79 | 0.68, 0.92 | 0.003 |
| 25-0HD (binary) |  |  |  |  |  |  |  |  |  |  |  |
| <10ng/ml | 136 | 829 | 1.00 |  |  | 1.00 |  |  | 1.00 |  |  |
| ≥10ng/ml | 156 | 1412 | 0.67 | 0.53, 0.86 | 0.002 | 0.71 | 0.56, 0.91 | 0.008 | 0.75 | 0.56, 0.99 | 0.04 |
| 25-0HD (quintiles) |  |  |  |  |  |  |  |  |  |  |  |
| <1.67 | 69 | 366 | 1.00 |  |  | 1.00 |  |  | 1.00 |  |  |
| 1.67-2.24 | 62 | 425 | 0.77 | 0.53, 1.12 | 0.18 | 0.77 | 0.53, 1.12 | 0.18 | 0.60 | 0.39, 0.92 | 0.02 |
| 2.24-2.58 | 67 | 430 | 0.83 | 0.57, 1.19 | 0.31 | 0.83 | 0.58, 1.21 | 0.33 | 0.76 | 0.51, 1.15 | 0.19 |
| 2.58-2.91 | 50 | 505 | 0.53 | 0.36, 0.77 | 0.001 | 0.54 | 0.36, 0.79 | 0.002 | 0.57 | 0.37, 0.87 | 0.009 |
| ≥2.91 | 42 | 511 | 0.44 | 0.29, 0.65 | <0.0005 | 0.49 | 0.79, 0.92 | 0.001 | 0.39 | 0.24, 0.63 | <0.005 |
| *p-value trend* |  |  |  |  | 8.1x10^-6^ |  |  | 0.0001 |  |  | 0.0003 |
| ***Only severe symptoms*** | *Cases* | *Controls* | *OR* | *95% CI* | *p-value* | *OR* | *95% CI* | *p-value* | *OR* | *95% CI* | *p-value* |
| 25-OHD  (continuous; ng/ml) | 220 | 2237 | 0.78 | 0.67, 0.90 | 0.001 | 0.78 | 0.67, 0.91 | 0.001 | 0.78 | 0.66, 0.94 | 0.008 |
| 25-0HD (binary) |  |  |  |  |  |  |  |  |  |  |  |
| <10ng/ml | 99 | 829 | 1.00 |  |  | 1.00 |  |  | 1.00 |  |  |
| ≥10ng/ml | 121 | 1412 | 0.72 | 0.54, 0.95 | 0.02 | 0.73 | 0.55, 0.97 | 0.03 | 0.72 | 0.52, 1.01 | 0.06 |
| 25-0HD (quintiles) |  |  |  |  |  |  |  |  |  |  |  |
| <1.67 | 57 | 366 | 1.00 |  |  | 1.00 |  |  | 1.00 |  |  |
| 1.67-2.24 | 39 | 425 | 0.59 | 0.38, 0.91 | 0.02 | 0.59 | 0.38, 0.91 | 0.02 | 0.50 | 0.30, 0.83 | 0.007 |
| 2.24-2.58 | 45 | 430 | 0.67 | 0.44, 1.02 | 0.06 | 0.67 | 0.44, 1.02 | 0.06 | 0.57 | 0.35, 0.94 | 0.03 |
| 2.58-2.91 | 46 | 505 | 0.58 | 0.39, 0.88 | 0.01 | 0.59 | 0.39, 0.89 | 0.01 | 0.54 | 0.33, 0.88 | 0.01 |
| ≥2.91 | 33 | 511 | 0.41 | 0.26, 0.65 | <0.0005 | 0.43 | 0.27, 0.67 | <0.0005 | 0.42 | 0.25, 0.71 | 0.001 |
| *p-value trend* |  |  |  |  | 0.0004 |  |  | 0.0007 |  |  | 0.004 |
| ***Both severe and mild symptoms**** | *Cases* | *Controls* | *OR* | *95% CI* | *p-value* | *OR* | *95% CI* | *p-value* | *OR* | *95% CI* | *p-value* |
| 25-OHD  (continuous; ng/ml) | 676 | 2237 | 0.76 | 0.69, 0.84 | 3.1x10^-8^ | 0.77 | 0.70, 0.85 | 9.7x10^-8^ | 0.74 | 0.67, 0.83 | 2.3x10^-7^ |
| 25-0HD (binary) |  |  |  |  |  |  |  |  |  |  |  |
| <10ng/ml | 340 | 829 | 1.00 |  |  | 1.00 |  |  | 1.00 |  |  |
| ≥10ng/ml | 338 | 1412 | 0.58 | 0.49, 0.69 | 1.1x10^-9^ | 0.59 | 0.50, 0.71 | 4.3x10^-9^ | 0.59 | 0.48, 0.72 | 2.4x10^-7^ |
| 25-0HD (quintiles) |  |  |  |  |  |  |  |  |  |  |  |
| <1.67 | 161 | 366 | 1.00 |  |  | 1.00 |  |  | 1.00 |  |  |
| 1.67-2.24 | 172 | 425 | 0.92 | 0.71, 1.19 | 0.52 | 0.92 | 0.72, 1.20 | 0.55 | 0.89 | 0.66, 1.20 | 0.45 |
| 2.24-2.58 | 117 | 430 | 0.62 | 0.47, 0.82 | 0.001 | 0.63 | 0.47, 0.82 | 0.001 | 0.67 | 0.49, 0.92 | 0.01 |
| 2.58-2.91 | 120 | 505 | 0.54 | 0.41, 0.71 | <0.0005 | 0.55 | 0.42, 0.72 | <0.0005 | 0.51 | 0.37, 0.70 | <0.0005 |
| ≥2.91 | 106 | 511 | 0.47 | 0.36, 0.62 | <0.0005 | 0.48 | 0.36, 0.64 | <0.0005 | 0.43 | 0.31, 0.61 | <0.0005 |
| *p-value trend* |  |  |  |  | 7.8x10^-11^ |  |  | 3.2x10^-10^ |  |  | 2.9x10^-9^ |

^*^ Including cases that presented with both, mild and severe symptoms at diagnosis
