# Supplementary material for: Instrumental Variable Estimation of the Causal Effect of Plasma 25-Hydroxy-Vitamin D on Colorectal Cancer Risk: A Mendelian Randomization Analysis
Source: PLoS One. 2012 Jun 6;7(6):e37662. doi: 10.1371/journal.pone.0037662 (PMC3368918; doi:10.1371/journal.pone.0037662)
Supplement: Table S5 — Logistic regression analysis for the association between plasma 25-0HD on colorectal cancer risk after stratification based on the time between diagnosis and recruittment (TDR). (DOC) [file pone.0037662.s005.doc]

Supplementary Table S5: Logistic regression analysis for the association between plasma 25-0HD on colorectal cancer risk after stratification based on the time between diagnosis and recruittment (TDR)

| **Standard logistic**  **regression analysis** | **N** | | **Crude model** | | | **Model I** | | | **Model II** | | |
| --- | --- | --- | --- | --- | --- | --- | --- | --- | --- | --- | --- |
| ***TDR ≤150 days*** | *Cases* | *Controls* | *OR* | *95% CI* | *p-value* | *OR* | *95% CI* | *p-value* | *OR* | *95% CI* | *p-value* |
| 25-OHD  (continuous; ng/ml) | 973 | 2237 | 0.77 | 0.71, 0.83 | 7.8x10^-5^ | 0.76 | 0.70, 0.83 | 2.97x10^-10^ | 0.73 | 0.66, 0.81 | 5.7x10^-10^ |
| 25-0HD (binary) |  |  |  |  |  |  |  |  |  |  |  |
| <10ng/ml | 465 | 829 | 1.00 |  |  | 1.00 |  |  | 1.00 |  |  |
| ≥10ng/ml | 509 | 1412 | 0.64 | 0.55, 0.75 | 8.7x10^-9^ | 0.63 | 0.54, 0.73 | 3.0x10^-9^ | 0.62 | 0.52, 0.74 | 2.3x10^-7^ |
| 25-0HD (quintiles) |  |  |  |  |  |  |  |  |  |  |  |
| <1.67 | 234 | 366 | 1.00 |  |  | 1.00 |  |  | 1.00 |  |  |
| 1.67-2.24 | 214 | 425 | 0.79 | 0.63, 1.00 | 0.05 | 0.80 | 0.63, 1.00 | 0.06 | 0.69 | 0.53, 0.90 | 0.007 |
| 2.24-2.58 | 181 | 430 | 0.66 | 0.52, 0.84 | 0.001 | 0.66 | 0.52, 0.84 | 0.001 | 0.61 | 0.46, 0.80 | <0.0005 |
| 2.58-2.91 | 191 | 505 | 0.59 | 0.47, 0.75 | <0.0005 | 0.59 | 0.46, 0.74 | <0.0005 | 0.52 | 0.40, 0.69 | <0.0005 |
| ≥2.91 | 154 | 511 | 0.47 | 0.37, 0.60 | <0.0005 | 0.46 | 0.36, 0.58 | <0.0005 | 0.41 | 0.31, 0.55 | <0.0005 |
| *p-value trend* |  |  |  |  | 1.1x10^-10^ |  |  | 2.0x10^-11^ |  |  | 3.7x10^-10^ |
| ***TDR >150 days*** | *Cases* | *Controls* | *OR* | *95% CI* | *p-value* | *OR* | *95% CI* | *p-value* | *OR* | *95% CI* | *p-value* |
| 25-OHD  (continuous; ng/ml) | 1026 | 2237 | 0.76 | 0.70, 0.83 | 1.3x10^-10^ | 0.76 | 0.70, 0.83 | 1.3x10^-10^ | 0.76 | 0.68, 0.84 | 7.0x10^-8^ |
| 25-0HD (binary) |  |  |  |  |  |  |  |  |  |  |  |
| <10ng/ml | 507 | 829 | 1.00 |  |  | 1.00 |  |  | 1.00 |  |  |
| ≥10ng/ml | 520 | 1412 | 0.60 | 0.52, 0.70 | 1.7x10^-11^ | 0.60 | 0.51, 0.69 | 1.7x10^-11^ | 0.60 | 0.50, 0.72 | 4.7x10^-8^ |
| 25-0HD (quintiles) |  |  |  |  |  |  |  |  |  |  |  |
| <1.67 | 252 | 366 | 1.00 |  |  | 1.00 |  |  | 1.00 |  |  |
| 1.67-2.24 | 246 | 425 | 0.84 | 0.67, 1.05 | 0.13 | 0.84 | 0.67, 1.05 | 0.13 | 0.80 | 0.61, 1.04 | 0.10 |
| 2.24-2.58 | 196 | 430 | 0.66 | 0.52, 0.83 | <0.0005 | 0.66 | 0.52, 0.83 | <0.0005 | 0.72 | 0.55, 0.94 | 0.02 |
| 2.58-2.91 | 167 | 505 | 0.48 | 0.38, 0.61 | <0.0005 | 0.48 | 0.38, 0.61 | <0.0005 | 0.43 | 0.33, 0.58 | <0.0005 |
| ≥2.91 | 166 | 511 | 0.47 | 0.37, 0.60 | <0.0005 | 0.47 | 0.37, 0.60 | <0.0005 | 0.44 | 0.33, 0.59 | <0.0005 |
| *p-value trend* |  |  |  |  | 2.1x10^-14^ |  |  | 2.2x10^-14^ |  |  | 1.8x10^-11^ |
