# Supplementary material for: Instrumental Variable Estimation of the Causal Effect of Plasma 25-Hydroxy-Vitamin D on Colorectal Cancer Risk: A Mendelian Randomization Analysis
Source: PLoS One. 2012 Jun 6;7(6):e37662. doi: 10.1371/journal.pone.0037662 (PMC3368918; doi:10.1371/journal.pone.0037662)
Supplement: Table S6 — Distribution of possible confounding factors and the instruments. (DOC) [file pone.0037662.s006.doc]

Supplementary Table S6: Distribution of possible confounding factors and the instruments

| **rs2282679** | **Total N** | **CC** |  | **AC** |  | **AA** |  | **p-value** |
| --- | --- | --- | --- | --- | --- | --- | --- | --- |
|  |  | **N** | **%** | **N** | **%** | **N** | **%** | **from χ^2^ test** |
| N and % of participants | 3831 | 298 | 7.78% | 1603 | 41.84% | 1930 | 50.38% |  |
| Male sex | 3831 | 170 | 57.05% | 932 | 58.14% | 1062 | 50.94% | 0.15 |
| Deprivation score ≤3 | 3830 | 178 | 59.73% | 893 | 55.74% | 1123 | 58.19% | 0.23 |
| Low family history risk | 3692 | 261 | 93.21% | 1408 | 91.13% | 1708 | 91.48% | 0.52 |
| 0 hours of cycling and other sport activities | 3735 | 130 | 50.98% | 788 | 56.53% | 933 | 55.94 | 0.26 |
| No smoking | 3856 | 225 | 83.64% | 1154 | 80.76% | 1414 | 82.07% | 0.43 |
| No NSAIDs intake | 3451 | 174 | 64.68% | 983 | 68.12% | 1161 | 66.76% | 0.48 |
|  | **Total N** | **CC** |  | **AC** |  | **AA** |  | **p-value** |
|  |  | **Mean** | **SD** | **Mean** | **SD** | **Mean** | **SD** | **from ANOVA** |
| Age | 3827 | 60.31 | 10.42 | 60.73 | 10.83 | 60.78 | 10.91 | 0.79 |
| BMI | 3412 | 26.56 | 4.58 | 26.73 | 4.54 | 26.85 | 15.82 | 0.55 |
| Dietary energy intake (kcals) | 3453 | 2490 | 851.22 | 2622 | 974.88 | 2609 | 1275.59 | 0.21 |
| Alcohol intake (g) | 3453 | 11.69 | 13.10 | 13.54 | 16.02 | 13.31 | 16.19 | 0.21 |
|  |  |  |  |  |  |  |  |  |
| **rs12785878** | **Total N** | **GG** |  | **TG** |  | **TT** |  | **p-value** |
|  |  | **N** | **%** | **N** | **%** | **N** | **%** | **from χ^2^ test** |
| N and % of participants | 3574 | 138 | 3.86% | 1090 | 30.50% | 2346 | 65.64% |  |
| Male sex | 3574 | 80 | 57.97% | 630 | 57.80% | 1324 | 56.44% | 0.73 |
| Deprivation score ≤3 | 3573 | 80 | 57.97% | 616 | 56.57% | 1352 | 57.63% | 0.83 |
| Low family history risk | 3445 | 123 | 90.44% | 973 | 92.84% | 2075 | 91.77% | 0.45 |
| 0 hours of cycling and other sport activities | 3126 | 72 | 58.06% | 525 | 54.63% | 1149 | 56.30% | 0.61 |
| No smoking | 3231 | 93 | 75.00% | 821 | 82.93% | 1753 | 82.81% | 0.08 |
| No NSAIDs intake | 3263 | 80 | 62.99% | 658 | 65.93% | 1433 | 67.03% | 0.57 |
|  | **Total N** | **GG** |  | **TG** |  | **TT** |  | **p-value** |
|  |  | **Mean** | **SD** | **Mean** | **SD** | **Mean** | **SD** | **from ANOVA** |
| Age | 3570 | 61.59 | 10.81 | 61.75 | 10.44 | 61.48 | 10.78 | 0.78 |
| BMI | 3226 | 27.00 | 4.68 | 26.72 | 4.58 | 26.76 | 4.55 | 0.82 |
| Dietary energy intake (kcals) | 3265 | 2749 | 976.17 | 2611 | 1043.6 | 2597 | 1181.3 | 0.47 |
| Alcohol intake (g) | 3265 | 15.51 | 19.20 | 13.31 | 15.17 | 13.06 | 15.93 | 0.24 |
| **rs10741657** | **Total N** | **GG** |  | **GA** |  | **AA** |  | **p-value** |
|  |  | **N** | **%** | **N** | **%** | **N** | **%** | **from χ^2^ test** |
| N and % of participants | 3323 | 1184 | 35.63% | 1610 | 48.45% | 529 | 15.92% |  |
| Male sex | 3323 | 691 | 58.36% | 905 | 56.21% | 277 | 52.36% | 0.07 |
| Deprivation score ≤3 | 3323 | 679 | 57.35% | 951 | 59.07% | 299 | 56.52% | 0.49 |
| Low family history risk | 3209 | 1043 | 91.49% | 1431 | 91.97% | 470 | 91.62% | 0.90 |
| 0 hours of cycling and other sport activities | 2917 | 585 | 56.14% | 781 | 55% | 249 | 54.73% | 0.82 |
| No smoking | 3009 | 880 | 81.94% | 1210 | 82.93% | 385 | 80.88% | 0.56 |
| No NSAIDs intake | 3036 | 718 | 66.24% | 999 | 67.68% | 307 | 64.50% | 0.41 |
|  | **Total N** | **GG** |  | **GA** |  | **AA** |  | **p-value** |
|  |  | **Mean** | **SD** | **Mean** | **SD** | **Mean** | **SD** | **from ANOVA** |
| Age | 3320 | 61.59 | 10.87 | 61.53 | 10.75 | 61.10 | 10.67 | 0.67 |
| BMI | 2999 | 26.73 | 4.60 | 26.78 | 4.52 | 26.98 | 4.68 | 0.59 |
| Dietary energy intake (kcals) | 3039 | 2614 | 987.4 | 2574 | 1268 | 2609 | 959.6 | 0.65 |
| Alcohol intake (g) | 3039 | 13.02 | 15.05 | 13.49 | 16.85 | 13.26 | 15.65 | 0.77 |
|  |  |  |  |  |  |  |  |  |
| **rs6013897** | **Total N** | **AA** |  | **TA** |  | **TT** |  | **p-value** |
|  |  | **N** | **%** | **N** | **%** | **N** | **%** | **from χ^2^ test** |
| N and % of participants | 3475 | 119 | 3.42% | 1069 | 30.76% | 2287 | 65.81% |  |
| Male sex | 3475 | 68 | 57.14% | 598 | 55.94% | 1292 | 56.49% | 0.94 |
| Deprivation score ≤3 | 3475 | 66 | 55.46% | 631 | 59.03% | 1307 | 57.15% | 0.52 |
| Low family history risk | 3354 | 103 | 89.57% | 953 | 92.52% | 2017 | 91.31% | 0.37 |
| 0 hours of cycling and other sport activities | 3030 | 58 | 54.21% | 505 | 54.18% | 1116 | 56.05% | 0.62 |
| No smoking | 3123 | 91 | 82.73% | 797 | 82.76% | 1692 | 82.54% | 0.99 |
| No NSAIDs intake | 3153 | 66 | 60.00% | 662 | 67.83% | 1372 | 66.38% | 0.24 |
|  | **Total N** | **AA** |  | **TA** |  | **TT** |  | **p-value** |
|  |  | **Mean** | **SD** | **Mean** | **SD** | **Mean** | **SD** | **from ANOVA** |
| Age | 3472 | 60.39 | 10.34 | 61.22 | 10.93 | 61.19 | 10.69 | 0.72 |
| BMI | 3118 | 27.15 | 5.10 | 26.79 | 4.44 | 26.81 | 4.62 | 0.74 |
| Dietary energy intake (kcals) | 3156 | 2535 | 935.2 | 2587 | 987.8 | 2609 | 1223 | 0.74 |
| Alcohol intake (g) | 3156 | 15.30 | 17.06 | 13.16 | 15.14 | 13.25 | 16.39 | 0.41 |
