# Supplementary material for: Instrumental Variable Estimation of the Causal Effect of Plasma 25-Hydroxy-Vitamin D on Colorectal Cancer Risk: A Mendelian Randomization Analysis
Source: PLoS One. 2012 Jun 6;7(6):e37662. doi: 10.1371/journal.pone.0037662 (PMC3368918; doi:10.1371/journal.pone.0037662)
Supplement: Table S7 — Wald/ratio instrumental variable estimator of the causal odds ratio for the effect of plasma 25(0H)D on colorectal cancer risk. (DOC) [file pone.0037662.s007.doc]

Supplementary Table S7: Wald/ ratio instrumental variable estimator of the causal odds ratio for the effect of plasma 25(0H)D on colorectal cancer risk

| **Model** | **plasma 25-0HD (continuous, ng/ml)** | | **F statistic** |
| --- | --- | --- | --- |
|  | *OR* | *95% CI* |  |
| *rs2282679* |  |  |  |
| Unadjusted | 0.58 | 0.11, 3.07 | 7.29 |
| Adjusted for age and sex | 0.94 | 0.49, 1.82 | 15.80 |
| *rs12785878* |  |  |  |
| Unadjusted | 3.94 | 0.92, 16.89 | 9.70 |
| Adjusted for age and sex | 1.22 | 0.60, 2.50 | 13.50 |
| *rs10741657* |  |  |  |
| Unadjusted | 1.15 | 0.00, 731.17 | 0.49 |
| Adjusted for age and sex | 0.89 | 0.40, 1.97 | 10.89 |
| *rs6013897* |  |  |  |
| Unadjusted | 2.23 | 0.06, 81.45 | 1.58 |
| Adjusted for age and sex | 0.99 | 0.40, 2.43 | 8.47 |
