# Supplementary material for: Instrumental Variable Estimation of the Causal Effect of Plasma 25-Hydroxy-Vitamin D on Colorectal Cancer Risk: A Mendelian Randomization Analysis
Source: PLoS One. 2012 Jun 6;7(6):e37662. doi: 10.1371/journal.pone.0037662 (PMC3368918; doi:10.1371/journal.pone.0037662)
Supplement: Table S8 — Two stage instrumental variable estimator of the causal odds ratio for the effect of plasma 25(0H)D on colorectal cancer risk. (DOC) [file pone.0037662.s008.doc]

Supplementary Table S8: Two stage instrumental variable estimator of the causal odds ratio for the effect of plasma 25(0H)D on colorectal cancer risk

| **Model** | **plasma 25-0HD (continuous, ng/ml)** | | **F statistic** |
| --- | --- | --- | --- |
|  | *coef.* | *95% CI* |  |
| *rs2282679* |  |  |  |
| Unadjusted | -21% | -54%, 13% | 1.48 |
| Adjusted for age and sex | -20% | -53%, 12% | 0.51 |
| *rs12785878* |  |  |  |
| Unadjusted | 26% | -5%, 56% | 2.68 |
| Adjusted for age and sex | 26% | -5%, 56% | 0.94 |
| *rs10741657* |  |  |  |
| Unadjusted | 65% | -162%, 291% | 0.31 |
| Adjusted for age and sex | 66% | -181%, 312% | 0.11 |
| *rs6013897* |  |  |  |
| Unadjusted | 31% | -66%, 128% | 0.40 |
| Adjusted for age and sex | 30% | -65%, 124% | 0.14 |
