# Supplementary material for: Instrumental Variable Estimation of the Causal Effect of Plasma 25-Hydroxy-Vitamin D on Colorectal Cancer Risk: A Mendelian Randomization Analysis
Source: PLoS One. 2012 Jun 6;7(6):e37662. doi: 10.1371/journal.pone.0037662 (PMC3368918; doi:10.1371/journal.pone.0037662)
Supplement: Table S9 — Multiplicative structural mean models instrumental variable estimator of the causal odds ratio for the effect of plasma 25(0H)D on colorectal cancer risk. (DOC) [file pone.0037662.s009.doc]

Supplementary Table S9: Multiplicative structural mean models instrumental variable estimator of the causal odds ratio for the effect of plasma 25(0H)D on colorectal cancer risk

| **Model** | **plasma 25-0HD (continuous, ng/ml)** | |
| --- | --- | --- |
|  | *RR* | *95% CI* |
| *rs2282679* |  |  |
| Unadjusted | 0.70 | 0.20, 2.39 |
| Adjusted for age and sex | 0.70 | 0.22, 2.29 |
| *rs12785878* |  |  |
| Unadjusted | 1.57 | 0.90, 2.73 |
| Adjusted for age and sex | 1.56 | 0.91, 2.69 |
| *rs10741657* |  |  |
| Unadjusted | 1.03 | 0.28, 3.73 |
| Adjusted for age and sex | 1.02 | 0.25, 4.22 |
| *rs6013897* |  |  |
| Unadjusted | 0.69 | 0.06, 8.20 |
| Adjusted for age and sex | 0.64 | 0.02, 19.54 |
