# Supplementary material for: Instrumental Variable Estimation of the Causal Effect of Plasma 25-Hydroxy-Vitamin D on Colorectal Cancer Risk: A Mendelian Randomization Analysis
Source: PLoS One. 2012 Jun 6;7(6):e37662. doi: 10.1371/journal.pone.0037662 (PMC3368918; doi:10.1371/journal.pone.0037662)
Supplement: Table S10 — Logistic structural mean models instrumental variable estimator of the causal odds ratio for the effect of plasma 25(0H)D on colorectal cancer risk. (DOC) [file pone.0037662.s010.doc]

Supplementary Table S10: Logistic structural mean models instrumental variable estimator of the causal odds ratio for the effect of plasma 25(0H)D on colorectal cancer risk

| **Model** | **plasma 25-0HD (continuous, ng/ml)** | |
| --- | --- | --- |
|  | *RR* | *95% CI* |
| *rs2282679* |  |  |
| Unadjusted | 0.60 | 0.16, 2.23 |
| Adjusted for age and sex | 0.62 | 0.18, 2.07 |
| *rs12785878* |  |  |
| Unadjusted | 2.25 | 1.00, 5.09 |
| Adjusted for age and sex | 2.16 | 0.95, 4.96 |
| *rs10741657* |  |  |
| Unadjusted | 1.08 | 0.04, 30.18 |
| Adjusted for age and sex | 1.03 | 0.04, 27.54 |
| *rs6013897* |  |  |
| Unadjusted | 21.99 | 0.13, 3848.34 |
| Adjusted for age and sex |  | not concave |
