# Supplementary material for: Instrumental Variable Estimation of the Causal Effect of Plasma 25-Hydroxy-Vitamin D on Colorectal Cancer Risk: A Mendelian Randomization Analysis
Source: PLoS One. 2012 Jun 6;7(6):e37662. doi: 10.1371/journal.pone.0037662 (PMC3368918; doi:10.1371/journal.pone.0037662)
Supplement: Table S11 — Review of the characteristics of the MR studies published in 2011. (DOC) [file pone.0037662.s011.doc]

Supplementary table S11: Review of the characteristics of the MR studies published in 2011.

| **Author[[1]](#endnote-2)** | **Phenotype** | **Outcome** | **Instrument** | **Size** | **Estimator** | **F statistic[[2]](#endnote-3)** | **Result** | **Direction of effect (IV)[[3]](#endnote-4)** | **Direction of effect (A)[[4]](#endnote-5)** | **Power**  **(estimated)[[5]](#endnote-6)** |
| --- | --- | --- | --- | --- | --- | --- | --- | --- | --- | --- |
| **Mumby HS (45)** | **High childhood BMI** | **Early menarche** | **BMI increasing allele score: FTO, MC4R, TMEM18, GNPDA2, KCTD15, NEGR1, BDNF, ETV5, MTCH2, SEC16B, FAIM2, SH2B1** | **8156** | **maximum likelihood estimator** | **not reported** | **statistically sign** | **+** | **+** | **~0.92** |
| Lawlor DA  (41) | High adiposity quantified by a) BMI and b) WHR | Psychological distress | FTO (rs9939609), MC4R (rs17782313) | 53221 | control function estimator | ranged from 5 to 85 | statistically sign | + | - | ~0.92 |
| Lewis SJ  (42) | Smoking | Depressed moood in pregnancy | CHRNA5-CHRNA3-CHRNB4 (rs1051730) | 6294 | two-stage least squares | not reported | not statistically sign | - | + | <0.68 |
| Pfister R  (39) | High uric acid levels | Type 2 diabetes | Genetic score of rs12129861, rs734553, rs2231142, rs742132, rs1165151, rs12356193, rs17300741, rs505802 | 16064 | MR meta-analysis | not reported | not statistically sign | Null | + | n/a |
| Wensley F  (37) | High CRP levels | Coronay heart disease | rs3093077, rs1205, rs1130864, rs1800947 | 194418 | MR meta-analysis | not reported | not statistically sign | Null | + | n/a |
| Breitling LP  (36) | High levels of type II Secretory Phospholipase A2 | Prognosis of coronary heart disease | PLA2G2A (rs4744, rs10732279) | 1014 | ratio estimator | not reported | not statistically sign | + | + | <0.18 |
| Benn M  (35) | Low levels of LDL cholesterol | Cancer | PCSK9 (rs11591174), ABCG8 (rs11887534), APOE (rs429358), R158C (rs7412) | 70179 | two-stage least squares | ranged from 23 to 844 | not statistically sign | Null | = | ~1.00 |
| **Kivimaki M**  **(44)** | **High levels of Lp(a)** | **Early atherosclerosis determined by: a) intima-media thickness (IMT) and b) branchial flow-mediated dilation (FMD)** | **rs783147** | **2080** | **two-stage least squares** | **269.6 for IMT and 446.0 for FMD** | **not statistically sign** | **Null** | **Null** | **~0.18** |
| **Kivimaki M**  **(40)** | **Long term obesity** | **Common mental disorders** | **FTO (rs1421085)** | **4145** | **two-stage least squares** | **not reported** | **statistically sign** | **+** | **+** | **~0.92** |
| Dahl M  (43) | High levels of CRP | Chronic obstructive pulomonary disease | rs3091244, rs1130864, rs1205, rs3093077 | 7974 | MR meta-analysis | not reported | not statistically sign | Null | + | n/a |
| De Silva NMG  (38) | Raised circulating triglyceride levels | Type 2 diabetes | weighted allele score of 12 SNPs | 8335 | control function estimator | not reported | statistically sign | - | + | ~0.92 |
|  | Raised circulating triglyceride levels | Fastin insulin, fasting glucose, HOMA-B, HOMA-IR | weighted allele score of 12 SNPs | 8271 | two-stage least squares | not reported | not statistically sign | Null | + | <0.10 |

1. Reference as appear in the main text are in brackets [↑](#endnote-ref-2)
2. Measures the strength of the instrument, with F>10 being an indication of a strong instrument [↑](#endnote-ref-3)
3. Direction of the effect from the instrumental variable analysis: +: the phenotype increases the risk of the outcome, -: the phenotype decreases the risk of the outcome, null: no causal effect [↑](#endnote-ref-4)
4. Direction of the effect from the conventions analysis (observational epidemiology) : +: the phenotype increases the risk of the outcome, -: the phenotype decreases the risk of the outcome, null: no association [↑](#endnote-ref-5)
5. Estimated using info published in Pierce BL et al, 2011 [↑](#endnote-ref-6)
