# Supplementary material for: Instrumental Variable Estimation of the Causal Effect of Plasma 25-Hydroxy-Vitamin D on Colorectal Cancer Risk: A Mendelian Randomization Analysis
Source: PLoS One. 2012 Jun 6;7(6):e37662. doi: 10.1371/journal.pone.0037662 (PMC3368918; doi:10.1371/journal.pone.0037662)
Supplement: Methods S1 — Information about the following Instrumental Variable estimators: Wald (ratio) estimator, Two stage least squares estimator, Multiplicative structural mean models, Logistic structural mean models. (DOC) [file pone.0037662.s012.doc]

**Supplementary methods**

To estimate the causal odds ratio (COR) we applied the additional four IV estimators for a 3-level categorical instrument Z coded 0, 1, 2 (SNP) a continuous intermediate phenotype X (plasma 25-OHD3) and a binary outcome Y (CRC).

*Wald (ratio) estimator*

The “Wald” (ratio) estimator the difference in the expected value of the outcome (Y) for a 1-unit difference in the intermediate phenotype (X) is defined as the ratio of the coefficients from a logistic regression of the outcome (Y) on the instrument(s) (Z) and of a linear regression of the intermediate phenotype (X) on the instrument(s) (Z) (1).

*Two stage least squares estimator*

The first stage of the two stage least squares is a linear regression of the intermediate phenotype (X) on the instrument(s) (Z), which generates predicted values for the intermediate phenotype. The second stage is a linear regression of the outcome (Y) on the predicted values of the intermediate phenotype (1). For a binary outcome two stage least squares estimator estimates a risk difference.

*Multiplicative structural mean models*

We used the multiplicative structural mean models to estimate the causal risk ratio and causal odds ratio of colorectal cancer for a unit increase in log vitamin D using the allele score as an instrumental variable. Structural mean models (SMMs) exploit instrumental variables via G-estimation, which involves finding the value of the causal parameter that fulfils the conditional mean independence assumption (2). The multiplicative SMM (MSMM) assumes a log-linear structural model for the effect of log vitamin D on colorectal cancer with no effect modification by the instrument (2). Note that the first stage regression is weighted for the case control status (3).

*Logistic structural mean models*

The logistic structural mean model (LSMM) assumes a logistic structural model for colorectal cancer given log vitamin D, with no effect modification by the instrument. We implemented the double logistic SMM of Vansteelandt and Goetghebeur (2003) (4) which fits an association model before the causal model because G-estimation cannot be performed in a single step for LSMM (1). Note that the first stage regression is weighted for the case control status (3).

References

1. Palmer TM et al. Instrumental variable estimation of causal risk ratios and causal odds ratios in mendelian randomization analyses. Am.J Epidemiol 2011;173:1392-403.

2. Hernan MA, Robins JM. Instruments for causal inference: an epidemiologist's dream? Epidemiology 2006;17:360-72.

3. Bowden J, Vansteelandt S. Mendelian randomization analysis of case-control data using structural mean models. Stat.Med 2011;30:678-94.

4. Goetghebeur E, Vansteelandt S. Structural mean models for compliance analysis in randomized clinical trials and the impact of errors on measures of exposure. Stat.Methods Med Res 2005;14:397-415.
